# Phase 1 First-in-Human Dose Escalation and Dose Expansion Study of KLS-1 (64Zinc Aspartate) in Patients With Cancer and Neurodegenerative Diseases

**DOI:** 10.7759/cureus.29921

**Published:** 2022-10-04

**Authors:** Jesus A Perez, Javier J Lopez, Claudia C Torres Badillo, Jaya Gill, Santosh Kesari, Peter Novak, Max Temnikov, Roman Byshovets, Oleg Bychkov

**Affiliations:** 1 Medicine, Pan American Cancer Treatment Center, Tijuana, MEX; 2 Neurology, Pan American Cancer Treatment Center, Tijuana, MEX; 3 General Practice, Pan American Cancer Treatment Center, Tijuana, MEX; 4 Department of Translational Neurosciences, Pacific Neuroscience Institute and Saint John’s Cancer Institute at Providence Saint John’s Health Center, Santa Monica, USA; 5 Physical Chemistry, Vector Vitale, North Miami Beach, USA; 6 Research, Vector Vitale, North Miami Beach, USA

**Keywords:** neuro-oncology, zinc, glioblastoma, amyotrophic lateral sclerosis, light isotopes, 64zinc aspartate, oncology clinical trials, supportive oncology, neurology and neuro-oncology, zinc aspartate

## Abstract

Background

KLS-1 is zinc (Zn) aspartate enriched with isotope ^64^Zn to 99.2% mass fraction of total zinc. KLS-1 is intended as a novel therapeutic approach for patients with a variety of diseases including but not limited to different forms of cancer and neurodegenerative diseases. The purpose of this first-in-human study was to determine the maximum tolerated dose (MTD), safety, and pharmacokinetics (PK) in patients with medical disorders.

Methods

The study was designed as consisting of two consecutive parts: the dose escalation part and the dose expansion part. Adult patients with refractory glioblastoma, primary progressive aphasia/dementia, amyotrophic lateral sclerosis, Parkinson’s disease (PD), and type 1 diabetes were included. KLS-1 formulated as a 10 mL water solution containing 26.42 mg/mL of ^64^zinc aspartate (that is equivalent to 5.184 mg/mL of ^64^Zn) was administered twice weekly in two-week cycles via two-hour intravenous (IV) infusion at various dose levels during the dose escalation part and twice weekly during five subsequent weeks in the dose expansion part. The study was conducted at Pan American Cancer Treatment Center (Tijuana, Mexico) in 2020 and had a duration of 10 months.

Results

A total of eight patients (all white/Caucasian) were enrolled in both parts of the study. A total of four patients who participated in the dose escalation part were dosed twice weekly at 1, 2, and 4 mg/kg in two-week cycles for each dose level with the dose increased to the next higher level in the subsequent cycle. Dose-limiting toxicities (DLTs) were defined at dose level 4 mg/kg due to treatment-emergent reversible adverse events that required medications for symptomatic relief. The most common drug-related toxicities that occurred in two or more patients (≥25%) were weakness (five patients), fatigue (four patients), dizziness (three patients), nausea (two patients), poor sleep (two patients), and abdominal discomfort (two patients). In the dose expansion part, a dose of 2 mg/kg administered twice weekly was investigated for five continuous weeks in four patients and was established as recommended phase 1b/2 dose. Systemic exposure to KLS-1 (area under the curve (AUC) and maximum serum concentration (C_max_)) increased from 1 to 4 mg/kg and showed a linear relationship.

Conclusions

Multiple doses of KLS-1 ranging from 1 to 2 mg/kg administered twice a week via intravenous infusion for up to five continuous weeks were safe and well tolerated in patients with different types of therapeutic conditions including but not limited to a few forms of cancer and Parkinson’s disease, and the evaluated pharmacokinetic parameters exhibited favorable profile.

## Introduction

One of the reasons why current medicines are not curative for the most challenging diseases is that the drugs do not target the underlying mechanism that engenders the onset of all pathologies. Since no reliable models of pathogenesis exist, most clinicians focus on the manifestations and consequences of pathologies in various cells within the human body. The significance of the subtle processes of the functioning of subcellular structures in many respects remains underestimated, and the mechanisms of the regulation of these processes are insufficiently studied.

Investigation of the role of amino acid chirality in proteins as a possible cause of dysfunction and changes in the ratio of light and heavy isotopes of essential elements can make an important contribution to understanding the basic triggers for the development of pathology and open conceptually new directions in therapy. Within a stable system, the presence of just one single D-amino acid in the structure of protein should be considered the first symptom of conformational disorder and the start of the protein’s degradation, which is commonplace in degenerative diseases, cancer, and atherosclerosis and distinctive for the general processes of aging. The violation of protein fidelity inevitably leads to the malfunction of the keylock mechanism recognition system. The outcome is a gradual or avalanche-like degradation of homeostasis, accompanied by the onset of symptoms related to autoimmune and metabolic disorders, the inability of the immune system to control and suppress the proliferation of cancer cells, neurodegenerative diseases, and other pathologies [1-3].

One of the possible mechanisms for regulating the correct ratio of L- and D-biomolecules is the modulation of the ratio between light and heavy isotopes of chemical elements [4]. The most promising element in this regard is zinc (Zn) as it plays an essential role in both transcription and translation processes. The problem is that the natural abundance of isotopes most often does not favor the appropriate ratio between protons and neutrons. The balance is quite good for the atoms of C, O, and N but unfortunately is not acceptable in most of the essential elements [5]. Together with food, water, and air, human beings get a mixture of stable natural isotopes, which become the building blocks for all biomolecules found in the human body, including polypeptides and proteins. When isotope substitution takes place in “inorganic materials,” it may lead to minor changes in the physical and chemical properties. However, the substitution of light isotopes with heavy ones in biomolecules may manifest the onset of pathological changes and often becomes a matter of life and death. Isotope substitution leading to the reversal of biomolecules’ “wrong” chirality has another side. Chirality amplification in autocatalytic reactions must be accompanied by the accumulation of light isotopes in healthy cells and heavy isotopes in pathology-affected cells, tissues, and organs [4,6].

The study of essential elements and their isotope ratios in various cells and hormones provides strong indications that the red bone marrow, organs, and cells producing signaling molecules and hormones are able to separate isotopes [7-9]. This makes it possible to enrich critical cells and molecules with light isotopes, to ensure that polypeptides and proteins have the normal conformation.

Zinc is recognized as one of the most essential elements for the human body [10-12]. Numerous scientific articles have documented the effect of excess or lack of zinc on human health. The influence of zinc is mentioned in relation to at least 60 diseases [13], including cancer [14]. Our hypothesis that the enrichment of organisms with light isotopes of zinc may be a kind of effective treatment for many medical disorders is built on the specific role of zinc in the synthesis of proteins and signal molecules. Common features in ribosomal proteins are zinc finger motifs; they have been associated with the proteins of both subunits of the ribosomes [15]. Ribosomal proteins play a major role as RNA-binding proteins. The success of the translation process hinges on the proper binding of zinc finger proteins and therefore on their conformation [16]. Ribosomes are rapidly produced in autocatalytic reactions. Autocatalytic reactions may amplify the yield of proteins with the wrong conformation induced by the isotope substitution of heavy isotopes. The isotope ratio for the stable isotope with a minimum excess of neutrons over protons ^64^Zn is just 48.60% [17]; therefore, the probability of substitution by heavier isotopes is very high. The situation could be negatively impacted by asymmetric autocatalysis. It renders the probability of the resulting mistranslation in all produced proteins very high. However, the most important issue is which precise zinc of the five stable isotopes, and it is not addressed in scientific literature. It is this issue that is fundamental to the proper operation of the ribosome and, therefore, for the synthesis of proteins with the correct conformation.

The KLS-1 drug substance is zinc aspartate enriched with isotope ^64^Zn to 99.2% mass fraction of total zinc. KLS-1 is formulated as a solution for intravenous (IV) infusions, containing 26.42 mg of drug substance in 1 mL of water for injections, which is equivalent to 5.184 mg of ^64^Zn.

The nonclinical pharmacology, pharmacokinetics (PK), and toxicology profiles of KLS-1 have been characterized in an extensive program of in vitro and in vivo studies. In these studies, KLS-1 has demonstrated visible pharmacodynamic activity and a favorable safety profile (our unpublished data). The data from these studies support the clinical development of KLS-1 as a novel therapy for a number of medical disorders and in particular different types of cancer, neurodegenerative diseases, aging diseases, and possibly many other that opens an avenue for further intensive clinical studies. This first-in-humans phase 1 study’s purpose was to evaluate the safety, determine the maximum tolerated dose (MTD), and investigate primary pharmacokinetic parameters of KLS-1 in patients with selected therapeutic disorders.

## Materials and methods

Study design

This was a phase 1, single-center, open-label study that consisted of two parts: a multiple-dose (MD) dose escalation part and an MD dose-expansion part. The dose escalation part utilized a 3+3 design that considered twice-a-week administration of KLS-1 in two-week cycles (four infusions per each cycle). The starting dose of KLS-1 of 1 mg/kg (equivalent to 0.2 mg/kg of ^64^zinc) was defined in preclinical on-animal studies. The intra-patient escalation was allowed in up to four successive cycles with a five-day interval between cycles. A safety committee reviewed the safety data for each dosing cohort prior to recommending the initiation of intra-patient dose escalation in a subsequent cycle.

In the dose expansion part, the selected dose of KLS-1 was administered twice a week for five weeks.

Patient eligibility

Eligible patients who were ≥18 years old with a histologically confirmed diagnosis of cancer (including brain cancers), neurological disorders (dementia, amyotrophic lateral sclerosis, Parkinson’s disease (PD), etc.), and metabolic disorders (including type 1 diabetes mellitus) participated in this study. All participants signed an informed consent form prior to enrollment in the study.

Subjects with cancer had progression on standard therapy, continued symptoms on current therapy, or were intolerant to therapy. Patients had a life expectancy of ≥8 weeks, adequate organ and marrow functions, and Karnofsky Performance Status (KPS) scores ≥ 60%. Patients were required to have recovered from the acute toxic effects of prior therapy and not to have received investigational agents within 28 days of study entry, cytotoxic therapy within 28 days, noncytotoxic agents within seven days, or surgery within four weeks.

Subjects with non-cancer diagnoses had progression on standard therapy. Patients had a life expectancy of ≥8 weeks, and adequate organ and marrow functions were maintained on their regular medical regimens.

The key exclusion criteria included a history of allergic reactions attributed to compounds of similar chemical or biologic composition, severe or uncontrolled concurrent medical disorders, impaired cardiac function, pregnancy, or nursing.

Approval by the Ethics Committee of Instituto de Medicina Regenerativa (Tijuana, Mexico) was obtained on October 1, 2019, and the study was conducted in accordance with the Declaration of Helsinki and the International Conference on Harmonization Good Clinical Practice Guidelines. Patients provided written informed consent.

Treatment regimen and dose escalation

The required volume of KLS-1 (calculated as per the patient’s body weight) was dissolved in 500 mL of normal saline (0.9% of NaCl) before IV infusion. In the dose escalation part of the study, KLS-1 was administered twice weekly via two-hour intravenous infusions in two-week cycles as defined above.

Definition of DLT and MTD

In the dose escalation phase, dose escalation, which followed a standard 3+3 design, was guided by assessing all grade toxicities and trends in adverse events seen in current and subsequent dosing cycles. If no DLT was identified at a certain dose level, intra-patient escalation was performed in subsequence dosing cycles until the maximum tolerated dose was defined. The starting dose of KLS-1 was 1 mg/kg (0.2 mg/kg of ^64^zinc) with the increase in subsequent cycles to 2 mg/kg (0.4 mg/kg of ^64^zinc) and 4 mg/kg (0.8 mg/kg of ^64^zinc).

In the dose expansion phase, the dose of 2 mg/kg (0.4 mg/kg of ^64^zinc) was investigated as the proposed phase 2 dose via twice-a-week administration for five subsequent weeks.

Dose-limiting toxicity (DLT) was defined as any adverse clinical event or clinically significant laboratory abnormality occurring during a treatment cycle that, in the opinion of the investigator, is (possibly or likely) related to the investigational medicinal product. Any grade 3 or 4 toxicity was DLT, including the toxicities listed as follows, which should be considered clinically significant: any grade 3 or 4 non-hematological toxicity will constitute a DLT, excluding alopecia or unpremeditated nausea/vomiting; grade 3 nausea, vomiting, or diarrhea lasting >24 hours despite standard prophylaxis and/or treatment; grade 4 diarrhea and vomiting of any duration; grade 3 febrile neutropenia (defined as ANC < 1,000/mm^3^ with a single temperature of >38.3°C or a sustained temperature of ≥38.3°C for more than one hour); grade 4 febrile neutropenia of any duration; grade 4 neutropenia lasting >5 days (defined as a neutrophil count of <500/mm^3^); grade 4 thrombocytopenia or thrombocytopenia with clinically significant bleeding (i.e., bleeding that requires blood or platelet transfusion or other medical intervention or that may cause disability or death, such as cerebral hemorrhage); grade 4 anemia of any duration; and any clinically significant toxicity that precludes the administration of the next scheduled dose beyond seven days will be considered a DLT, or dose reduction for any reason.

MTD was defined as the maximum dose level at which less than 33% of patients experienced study drug-related DLTs.

Safety

Vital signs and monitoring for infusion reactions were performed within 30 minutes after the initiation of KLS-1 administration, at the end of the administration, and then at least one hour post-dose. Safety evaluations of hematology, clinical chemistry, and urinalysis were conducted weekly in cycles 1, 2, and 3 and every two weeks thereafter within two months. ECGs assessed during treatment were recorded prior to KLS-1 administration and within one hour after the end of study drug administration. Toxicities were assessed using the National Cancer Institute Common Terminology Criteria for Adverse Events (NCI CTCAE) version 4.03.

Pharmacokinetic analysis

Pharmacokinetic (PK) parameters were assessed as secondary endpoints to guide the optimal dose of KLS-1 and could be considered as preliminary PK data that are to be reevaluated in the following clinical studies. In cycles 1, 2, and 3 of the dose escalation phase, blood samples (5 mL) were collected before the KLS-1 dosing and at 0.5, one, two, four, and eight hours after the end of infusion on day 1 of the same cycle, before dosing, and one hour after the end of infusion on day 10 of cycles 1 and 2 and day 3 of cycle 3 to define the accumulation of KLS-1.

Taking into consideration the complexity of isotope ^64^Zn determination, the concentrations of total zinc in serum were evaluated using a validated method of atomic absorption at Laboratorio de Asesoria y Servicio Referido (Mexico City, Mexico). This approach was based on the assumption that the sharp increase of serum zinc concentration is caused overwhelmingly by IV infusion of KLS-1 in comparison with the theoretical increase due to the normal intake (with the food) that may cause some plain increase. In any way, specific ^64^Zn determination cannot separate the sources of this intake, since the part of ^64^Zn in natural abundance is still significant and constitutes 48.63% of natural zinc that comes into a body, e.g., with food. Therefore, the availability of the evaluation of total zinc serum concentration for PK analysis was targeted because the intake of zinc into the organism occurred mainly due to the intravenous administration of KLS-1, while other sources of zinc intake into the body (food) can be dismissed due to the fact that the theoretical intake with food (via absorption in the gastrointestinal tract) in any case was several times lower than intake as part of KLS-1, and the total zinc concentration in blood plasma defined at pre-dose was a baseline for further PK measurements. Non-compartmental analysis with a serial sampling design was used to calculate the key PK parameters. The area under the curve (AUC) was calculated using the linear trapezoidal method on measured concentrations and actual time points.

## Results

Patient characteristics

Between January and September 2020, a total of eight patients were enrolled in the study: four were included in the dose escalation cohort and four in the dose expansion cohort (Table 1). One patient at 1 mg/kg was non-evaluable having withdrawn early (after three dosing in cycle 1) for reasons unrelated to study treatment.

**Table 1:** Baseline patient demographics and characteristics

Characteristic	Number of patients		%
Median age, years (range)		61 (22-86)	
Males (n)	3		37.5
Females (n)	5		62.5
Race: white (n)	8		100
Diagnosis: dose escalation part			
Refractory glioblastoma (n)	2		25
Amyotrophic lateral sclerosis (n)	1		12.5
Primary progressive aphasia/dementia (n)	1		12.5
Diagnosis: dose expansion part			
Diabetes, type 1 (n)	1		12.5
Parkinson’s disease (n)	3		37.5

**Figure 1:**
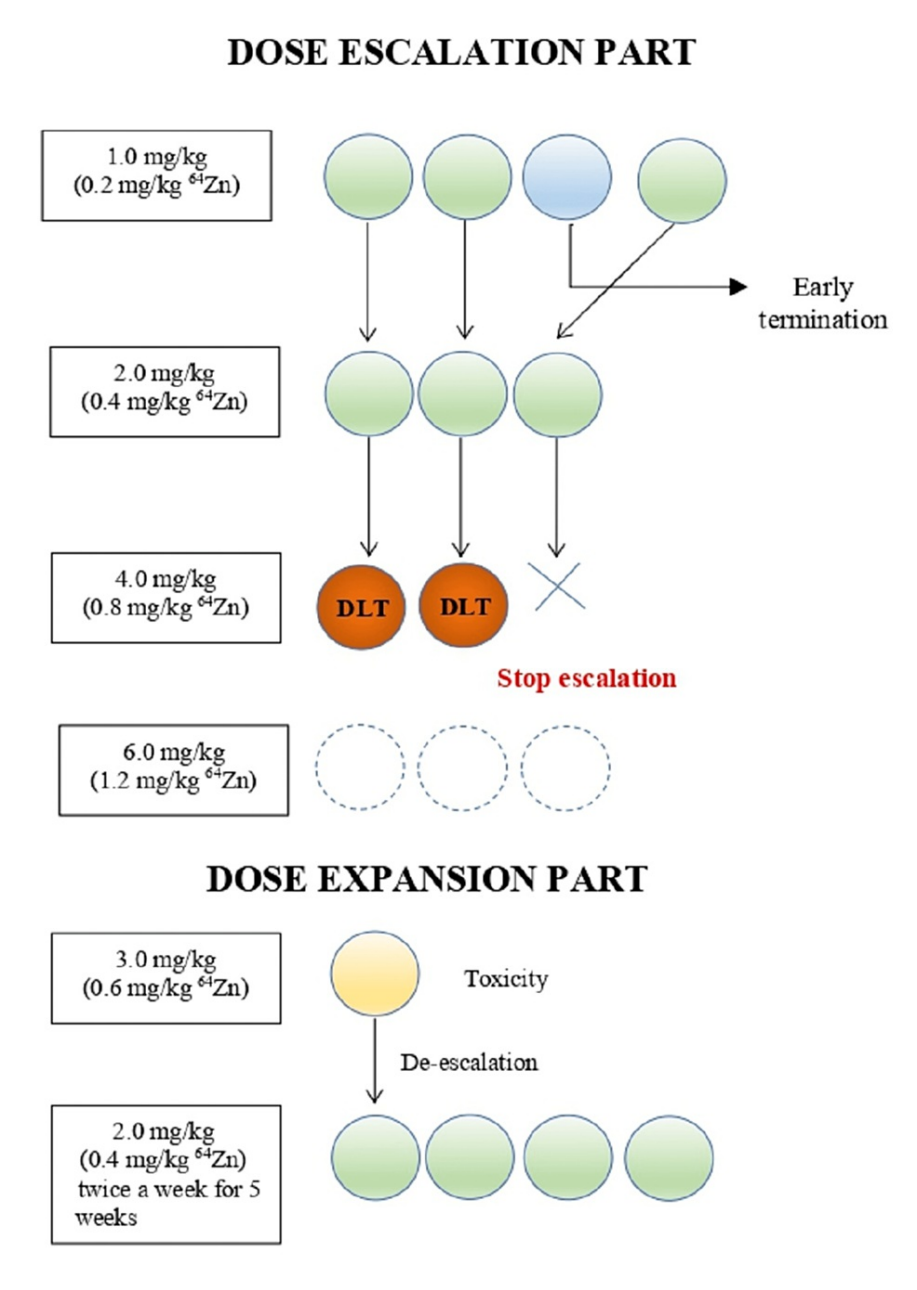
Patient distribution in the study Zn: zinc; DLT: dose-limiting toxicity

A general scheme of the study design and patients’ disposition in this study is outlined in Figure 1.

Dose escalation part

All patients in the dose escalation phase started treatment at a dose level of 1 mg/kg (0.2 mg/kg of ^64^zinc), which was administered twice a week on days 1, 3, 8, and 10. At this dose level, the tolerability of the study drug was quite good; no DLT was observed. Only one patient experienced mild signs of toxicity, in particular, slight fatigue during infusions and a mild headache, which could be associated with a slight increase in blood pressure during infusions. One more female patient early discontinued the study (via consent withdrawn), which was explained by the anxiety attack that happened on day 8 and was associated by the patient with the infusion of the study drug. This patient had a history of similar anxiety attacks in the past that had been associated with another treatment that she received. Patients showed no deviations from baseline in hematological, clinical chemistry parameters, urinalysis, and ECG. Finally, three patients completed the treatment in cycle 1 and were eligible for the dose increase in the second treatment cycle.

Cycle 2 considered intra-patient dose escalation up to 2 mg/kg (0.4 mg/kg of ^64^zinc). The study drug was administered on days 1, 3, 8, and 10 of this cycle to three patients who completed cycle 1 as described above. The tolerability of the study drug was satisfactory, and no signs of DLT were observed. Two patients complained of slight weakness and fatigue. Patients showed no deviations from baseline in hematological, clinical chemistry parameters, urinalysis, and ECG. The interesting finding in this cycle was that the patient with primary progressive aphasia had improvement in speech that was observed at the end of the cycle and lasted for several days. All patients fully completed this cycle and were eligible for the dose increase in the third treatment cycle.

Intra-patient dose escalation in cycle 3 considered the administration of KLS-1 at a dose of 4 mg/kg (0.8 mg/kg of ^64^zinc). Two patients already after the first administration of the study drug (day 1) complained of abdominal discomfort, nausea, severe weakness, dizziness, chills, and burning at the infusion site. Still, these signs did not reach the grades appropriate for DLT. After the second injection on day 3, these complaints persisted, and some of them intensified, reaching the grade 3 (in particular nausea lasting >24 hours despite standard treatment) that were appropriate for DLT. Therefore, further administration of the drug at a dose level of 4 mg/kg was stopped due to the manifestation of DLT in two out of three patients. The investigation of hematological, clinical chemistry parameters, urinalysis, and ECG still did not show any dramatical deviation from the baseline that could be interpreted as drug related, including the signs of DLT.

Dose expansion part

The second part of the study, the dose expansion part, considered twice-a-week administration of KLS-1 for five weeks at a dose level of 2 mg/kg (0.4 mg/kg of ^64^zinc). Four patients in total (three with PD and one with type 1 diabetes) were enrolled in the dose expansion cohort.

It should be noted that after analyzing the first part of the study and taking into account the pharmacokinetic parameters obtained in the dose escalation part, it was decided to test the dose of 3 mg/kg (0.6 mg/kg of ^64^zinc) as possibly safe. Therefore, the first patient who was included in this part of the study began treatment with a dose of 3 mg/kg. However, after the first dosing, this patient experienced severe nausea, which required the use of special medicines and still lasted more than 24 hours. It was decided to change the dosing regimen from twice a week to once a week; however, after the second and third injections, this symptom recurred.

Therefore, the decision was made to discontinue the study drug administration at a dose of 3 mg/kg and de-escalate the dose to 2 mg, which was confirmed as safe in the dose escalation part of the study.

Further, this and all other patients received KLS-1 at a dose of 2 mg/kg (0.4 mg/kg of ^64^zinc) in the dose expansion part.

Analyzing the data obtained from the dose expansion cohort, it can be concluded that during the entire period of treatment (five weeks), the study drug KLS-1 was well tolerated; no signs of severe toxicity were observed. Some patients experienced mild dizziness and fatigue and poor sleep, but these symptoms did not require medical intervention. Clinically significant deviation from the baseline in hematology, clinical chemistry, urinalysis, and ECG was not found.

The evaluation of the efficacy of KLS was not among the key objectives in this study; still, some preliminary signs of activity of KLS-1 were noted. Among the most interesting findings in the dose expansion part were the ability to walk more than usual (with the help of a walker), higher energy levels than usual, improved cognition, improved mood and speech, improved mental clarity, and improved balance and walking ability in patients with Parkinson’s disease. The patient with diabetes mellitus had better blood sugar control with a lower-than-usual requirement for basal insulin.

Safety and tolerability

The presence of adverse events and signs of toxicity of KLS-1 generally had a dose-dependent trend. Table 2 describes the occurrences of different types of adverse events and toxicity signs at different dose levels.

**Table 2:** Number of patients with treatment-related adverse events by the dose level of KLS-1

Adverse events/toxicity signs	Dose level (as per ^64^zinc)			
	0.2 mg/kg (n)	0.4 mg/kg (n)	0.6 mg/kg (n)	0.8 mg/kg (n)
Fatigue	2	4	1	-
Weakness	2	5	1	2
Dizziness	2	3	1	2
Headache	1	-	-	-
Chills	-	-	-	1
Pain in the site of injection	-	-	-	1
Poor sleep	-	-	1	2
Nausea	-	-	1	2
Abdominal discomfort	-	-	-	2

Upon reaching a dose of 4 mg/kg (0.8 mg/kg of ^64^zinc), patients experienced severe toxicity that occurred after the first and the second infusions and was interpreted as DLT, which caused a stop to further administration of the study drug.

At the same time, the dose of KLS-1 at 2 mg/kg (0.4 mg/kg of ^64^zinc) was well tolerated. All seven patients who were treated with this dose, both in the dose escalation and dose expansion parts, had no significant adverse events and signs of severe toxicity, and the complaints that occurred did not require any medical treatment.

All patients, seven in total (excluding one who terminated early), were followed up via survival contacts four months after the end of treatment, and no deaths were registered.

Pharmacokinetics of KLS-1

Pharmacokinetic data were obtained to guide the optimal dose of KLS-1. Blood samples (5 mL) were collected before the study drug dose and at 0.5, one, two, and eight hours after the end of infusion on day 1 of cycle 1. To evaluate the cumulative effect of KLS-1, additional samples were collected before and within 60 minutes after the end of infusion on day 10 in cycle 1 and cycle 2 and within 60 minutes after the end of infusion on day 3 in cycle 3. PK parameters are shown in Table 3.

**Table 3:** Summary of pharmacokinetic parameters after dosing with KLS-1 CV%: coefficient of variation; C_max_: maximum serum concentration; AUC: area under the curve; t 1/2: half-life time; Cl: clearance

Dose level (^64^Zn) mg/kg	Dose number	n	Geometric mean (CV%)			
			C_max_ (µg/mL)	AUC (µg hour /mL)	t _1/2_ (hour)	Cl (L/hour)
0.2	4	3	1.62 (29)	5.15 (6.45)	1.0 (36.9)	2.52 (3.91)
0.4	4	3	3.13 (17.47)	12.29 (11.22)	1.01 (29.45)	2.20 (16)
0.8	2	2	4.42 (16.84)	20.60 (8.43)	0.95 (26.95)	2.30 (14)

KLS-1 demonstrated quick and dose-independent elimination. The observed half-life (calculated from zinc plasma concentration) ranged from 0.72 to 1.42 hours. It was found that day 1’s plasma concentration of zinc at eight hours post-dose was below the baseline (pre-dose level) that was clearly visible in cycle 1 and cycle 2 and was not natural in cycle 3. It was defined as slight drug cumulative effect reflected in the increase of zinc plasma concentration at pre-dose on day 10 (ranging from 5% to 30%).

## Discussion

Since the further scope of clinical investigation of KLS-1 as a novel therapeutic concept involves studies in the areas of cancer and neurodegenerative diseases, patients with such pathologies have been targeted for this study, and that is acceptable for phase 1 trials. The results of our earlier preclinical on-animal studies support this choice of pathologies and have sourced the dose selection and the utilized schedule of the study drug administration.

Zinc and aspartate-containing compounds including zinc aspartate (having the natural zinc isotope abundance) are regular components of food supplements and parenteral nutrients in which safety is well established [18]. Any significant differences in the toxicological profile of zinc-containing compounds with the natural isotope abundance and ones enriched with the light ^64^Zn isotope were not expected since the chemical and essential biological properties are common for the different natural isotopes of chemical elements. Thus, the most expected adverse reactions of KLS-1 administration were nausea and vomiting, epigastric pain, abdominal cramps, diarrhea, anemia, and dizziness [19]. Among the expected reactions, only dizziness was reported often; at the same time, the other two most common complaints of patients in our study were fatigue and weakness, which were not much expected as per literature data. All the other adverse reactions were occasional and not systemic. It should be noted here that the adverse reactions for zinc compounds present in the literature are described when it is taken orally, while data for intravenous administration in humans are not available. Therefore, the high frequency of such adverse reactions as fatigue and dizziness may be associated with the intravenous administration of zinc aspartate.

Although phase 1 trials are usually limited to dose safety investigations and the scientific relevance of the results of these studies is usually limited, a couple of findings in this study are worth discussing.

As it is stated above, the eight hours post-dose zinc plasma concentrations were clearly lower than the baselines; this was found in cycles 1 and 2, but not in cycle 3. It was defined for day 1 infusions since only this administration was eligible for PK analysis. The nature of this finding can be explained that the intravenous infusion of light ^64^Zn isotope may either trigger zinc intracellular transport from blood or activate the increased elimination of zinc from the body. Further investigation of this phenomenon shall consider a more frequent schedule of PK investigation (e.g., during the third or fourth infusion and quantitative evaluation of zinc elimination with urine).

Another finding, i.e., the increase of the pre-dose zinc plasma concentration on days following day 1, in which appropriate measurements were done, may be explained as a zinc plasma cumulative effect, but this must be interpreted with caution due to the short observation window. The biological significance of this finding is not entirely clear, but most likely, this occurs due to the saturation of the body with a light zinc isotope and determines the therapeutic effect of KLS-1.

Unfortunately, the isotope analysis of zinc in blood plasma is technically very complex and, therefore, has not been utilized in this study and can hardly be applied in subsequent studies. Therefore, it is difficult to determine exactly which zinc isotope is responsible for the zinc plasma level decrease (below the pre-dose baselines) until the nearest eight hours after the injections and what is the pre-dose’s zinc isotope spectrum in the blood in the consistent administrations during the treatment cycle. However, we believe that the light zinc isotope displaces heavy isotopes, changing the intracellular zinc isotope spectrum with a shift in its favor, and thus, ^64^Zn becomes predominantly available, which determines the biological effect of KLS-1.

The defined improvement of the status of patients with PD and type 1 diabetes needs a larger observation window, and statistical confirmation of these observations, which was not the purpose of this study, thus has to be taken into account but still should be interpreted with caution.

## Conclusions

KLS-1 is generally well tolerated and safe at dose levels of 1 and 2 mg/kg. No signs of toxicity were defined based on patient examination, investigation of vital signs, and evaluation of laboratory parameters (hematology, clinical chemistry, and urinalysis) at these dose levels. DLT was defined at the dose level of 4 mg/kg (0.8 mg/kg of ^64^zinc). MTD was defined as 2 mg/kg (0.4 mg/kg of ^64^zinc) in the dose escalation phase and was confirmed in the dose expansion phase. This dose is recommended for further phase 1b/2 studies.

Interesting findings have been made that may be recognized as signs of preliminary activity of KLS-1 in patients with PD and type 1 diabetes that is worth to be further investigated in future studies.

The initial PK data obtained in this study confirm the quick elimination of KLS-1 at all tested doses that support safety data.
