# Correction: Phase 1 First-in-Human Dose Escalation and Dose Expansion Study of KLS-1 (64Zinc Aspartate) in Patients With Cancer and Neurodegenerative Diseases

**DOI:** 10.7759/cureus.c78

**Published:** 2022-10-14

**Authors:** Jesus A Perez, Javier J Lopez, Claudia C Torres Badillo, Jaya Gill, Santosh Kesari, Peter Novak, Max Temnikov, Roman Byshovets, Oleg Bychkov

**Affiliations:** 1 Medicine, Pan American Cancer Treatment Center, Tijuana, MEX; 2 Neurology, Pan American Cancer Treatment Center, Tijuana, MEX; 3 General Practice, Pan American Cancer Treatment Center, Tijuana, MEX; 4 Department of Translational Neurosciences, Pacific Neuroscience Institute and Saint John’s Cancer Institute at Providence Saint John’s Health Center, Santa Monica, USA; 5 Physical Chemistry, Vector Vitale, North Miami Beach, USA; 6 Research, Vector Vitale, North Miami Beach, USA

Authors Jaya Gill and Santosh Kesari have been added to the published article after they were mistakenly omitted from the original submission by the submitting author. The authors deeply regret this error.

 

